# Interleaved Acoustic Environments: Impact of an Auditory Scene Classification Procedure on Speech Perception in Cochlear Implant Users

**DOI:** 10.1177/23312165211014118

**Published:** 2021-05-24

**Authors:** Anja Eichenauer, Uwe Baumann, Timo Stöver, Tobias Weissgerber

**Affiliations:** Audiological Acoustics, ENT Department, University Hospital Frankfurt, Goethe University Frankfurt, Frankfurt am Main, Germany

**Keywords:** cochlear implants, speech perception, reverberation, automatic scene classification, room simulation

## Abstract

Clinical speech perception tests with simple presentation conditions often overestimate the impact of signal preprocessing on speech perception in complex listening environments. A new procedure was developed to assess speech perception in interleaved acoustic environments of different complexity that allows investigation of the impact of an automatic scene classification (ASC) algorithm on speech perception. The procedure was applied in cohorts of normal hearing (NH) controls and uni- and bilateral cochlear implant (CI) users. Speech reception thresholds (SRTs) were measured by means of a matrix sentence test in five acoustic environments that included different noise conditions (amplitude modulated and continuous), two spatial configurations, and reverberation. The acoustic environments were encapsulated in a randomized, mixed order single experimental run. Acoustic room simulation was played back with a loudspeaker auralization setup with 128 loudspeakers. 18 NH, 16 unilateral, and 16 bilateral CI users participated. SRTs were evaluated for each individual acoustic environment and as mean-SRT. Mean-SRTs improved by 2.4 dB signal-to-noise ratio for unilateral and 1.3 dB signal-to-noise ratio for bilateral CI users with activated ASC. Without ASC, the mean-SRT of bilateral CI users was 3.7 dB better than the SRT of unilateral CI users. The mean-SRT indicated significant differences, with NH group performing best and unilateral CI users performing worse with a difference of up to 13 dB compared to NH. The proposed speech test procedure successfully demonstrated that speech perception and benefit with ASC depend on the acoustic environment.

Speech perception in complex acoustic environments with interfering background noises and reverberation is demanding, even for people with normal hearing (NH). Although many cochlear implant (CI) users obtain high scores of speech perception in quiet, speech perception in the presence of additional competing sounds is often severely limited. Depending on the characteristics of the acoustic environment, target speech is masked and distorted in its spectral and temporal content, which reduces speech perception. However, conventional speech audiometry tests are usually performed with one or two loudspeakers, so that acoustic environments with different disturbing sounds from multiple directions are not reproduced. Furthermore, the influence of room acoustics on the effectiveness of signal preprocessing on speech perception is neglected in such test setups (Compton-Conley et al., 2004). Consequently, the challenges that individuals with hearing loss face in perceiving speech through different acoustic listening conditions have been poorly captured in routine clinical audiology.

Speech perception and the effect of signal processing on speech perception in acoustic environments with reverberation are of great interest. Loudspeaker-based acoustic room simulation is able to present complex acoustic conditions with controlled and reproducible setups; for example, Revit et al. (2007) developed an eight-channel sound reproduction system for real-world laboratory measurements (R-Space) into which microphone recordings are fed. Other loudspeaker-based room simulation systems use computer-based room modeling with determination of early reflections (Favrot, 2010; Grimm et al., 2015; Seeber et al., 2010). With this method, reverberation and multiple sound sources can be represented in a laboratory using multichannel loudspeaker arrangements. As an example for this approach, Kressner et al. (2018) applied a loudspeaker-based auralization system to simulate an acoustic environment with reverberation and evaluated speech perception in CI users. The authors reported deteriorated speech perception with CI aided participants as reverberation and source-receiver distance increased. The results demonstrate that reverberation plays an important role in speech perception which should be considered in audiological tests.

The improvement of speech perception in complex listening situations with reverberation has been the focus of hearing system development for years. However, most studies assessing the effect of signal preprocessing on speech perception have been conducted under free field (FF) listening conditions. For example, the beneficial effect of beamformers on speech perception in CI users has been shown in several studies with static or moving noise sources (Büchner et al., 2014; Honeder et al., 2018; Spriet et al., 2007; Weissgerber et al., 2015, 2017). Ricketts (2000) investigated the impact of beamformers in hearing aids in two listening environments with different reverberation times. They found significant decrease in speech perception benefit with activated hearing aid beamformers compared to omnidirectional signal transmission when reverberation increased. It was shown that test setups with a single, arbitrarily placed noise source provide large benefit with activated beamforming, but these results are not very representative for many listening situations.

Typically, the spatial sensitivity characteristic of the microphone is set by the audiologist for each listening program of the audio processor. Therefore, CI users have to select and activate the appropriate program manually to receive the benefit of beamforming. However, many CI users tend to always use their standard program (McMillan et al., 2018). For this reason, the setting of the CI processor is often not the best for a certain listening condition. Thus, speech perception is not enhanced to the technically possible extent in the presence of background noise. Automated scene classification (ASC) which is linked to an automated program selection can help to overcome this issue. The ASC classifies the acoustical environment by extraction and analysis of acoustic features from which the most likely listening condition is selected. The processor activates the beamformer setting considered as most suitable for the acoustic environment (Dorman & Natale, 2019; Mauger et al., 2014). Previous studies (Dorman & Natale, 2019; Mauger et al., 2014) reported significant improvements in speech perception in CI users with activated ASC algorithm. However, these evaluations were performed in constant FF conditions with sequential testing of certain spatial noise configurations. Up to now, it is not known whether the previously reported benefit of ASC in CI users can be reproduced in changing complex acoustic environments (e.g., in the presence of reverberation). This issue involves the accuracy of the classification of the listening environments as well as the benefit in speech perception with activated ASC.

For this purpose, the present work proposes a variant of the German matrix sentence test with interleaved acoustic environments (IAEs) of varying complexity that include reverberation. It provides a new test method to investigate the benefit of ASC on speech perception in a setup at which ASC permanently has to classify the presented acoustic environment and switch between listening programs. This so-called IAE method allows for analyzing the impact of ASC on speech reception thresholds (SRTs) of the acoustic environments, both, separately and in form of a combined Mean-SRT. The IAE test enables the assessment of multilayered speech perception results in only one test run with a rapid and condensed test design. Acoustic environments with reverberation were generated by means of computer-based room modeling with a multi-channel setup with 128 loudspeakers to implement acoustic room simulation.

It is hypothesized that ASC is able to adjust to the changing acoustic environments in the IAE setup and automatically selects a beneficial listening program. Compared to the standard program, there should exist a benefit of automated program selection by ASC on speech perception in CI users in all listening environments of the IAE setup. The overall Mean-SRT should reflect the detrimental effects of complex noise stimuli on speech perception in CI users. Given that, a comprehensive mean value for listening conditions of varying complexity is represented by Mean-SRT. In addition, the analysis of the SRTs of each acoustic environment shall reveal that type of noise and reverberation can have different effects on the test groups of NH persons and CI users.

## Methods

### Room Modeling and Simulation

**Figure 1.**
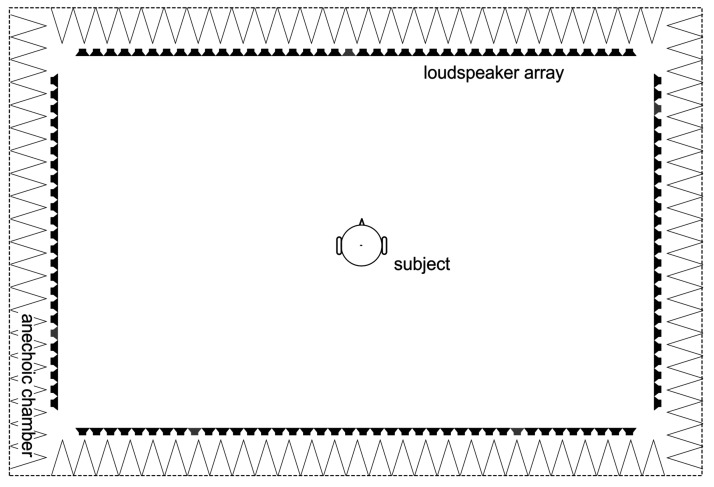
Sketch of the Laboratory With Listener, the Speakers Representing the Sound Sources (Direction of Direct Sounds) Are Marked.

**Figure 2.**
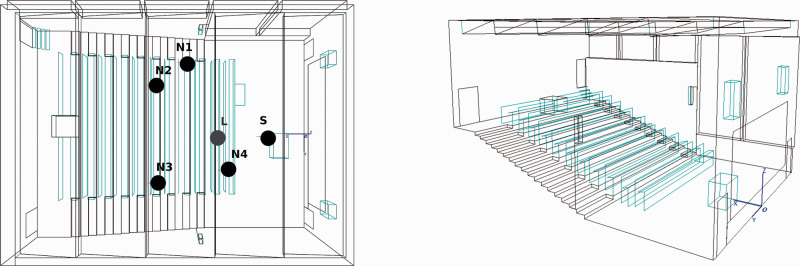
Sketch of the auditorium used for room simulation. Left: Room model of the auditorium with listener/receiver (L) and sound sources (S) for speech, N1, N2, N3, N4 for noise playback. Right: Side view of the room with staircase and benches, in the front of the room a lectern, loudspeakers and a blackboard are visible.

An anechoic chamber (4.10 × 2.60 × 2.10 m) equipped with 128 loudspeakers was used for sound reproduction. The loudspeakers are arranged in a rectangular shape in the horizontal plane (Figure 1). This setup was already used for speech tests under FF conditions (Weissgerber et al., 2015, 2017; 2019) or for the simulation of diffuse noise (Weissgerber et al., 2016). A three-dimensional room model of an auditorium was used for the room acoustic calculations (Figure 2). The auditorium had a volume of 1417 m^3^ and was designed with realistic absorbance and reflection properties of surfaces such as walls and furniture. The assigned material for the stairs was wood, the ceiling was lined with panels with slots of mineral wool, and the windows have the absorbance properties of glass.

The modeling of room acoustics was carried out with the software ODEON 14.01 (ODEON A/S, Lyngby, Denmark). The estimated mean reverberation time (RT60) of the room was 0.42 s. The distinctness (D50) was 42%, which describes that 42% of the total energy occurs within the first 50 ms after the direct sound.

In the modeled room, a receiver (i.e., the listener) was placed in the second row of the auditorium, surrounded by five sound sources. The source in front of the receiver (at 0°) was used as the target speech source. Four noise sources were placed asymmetrically around the receiver (65°, 135°, 225°, and 255°). In the room model, the distance between speech source and receiver was 3.15 m.

Using ODEON, the propagation of sound waves in the entire room for each combination of receiver and sound source was calculated using advanced ray-tracing algorithms. Early reflections up to an order of 10 were computed and extracted as a reflectogram. The generated reflectogram includes sound pressure levels in octave bands, temporal delays relative to direct sound, and azimuth and elevation angle of each reflection. The reflectograms were imported to MATLAB (The Mathworks, Natick, USA) for further processing. Considering the sound pressure levels of each octave band, a linear-phase finite impulse response (IR) filter was generated for each reflection and the respective temporal delay was added. Each reflection with its individual spectral shape and temporal delay was then mapped to the nearest loudspeaker in the laboratory according to its azimuth angle. Late reflections were generated using a feedback-delay network (obtained from the Quality and Usability Lab at Technical University Berlin). A frequency-dependent signal was generated for each of the 128 channels based on the reverberation time. Late reflections were faded in 40 ms after the direct sound using a Hann window with a 10 ms ramp and adjusted in level at point of insertion. To compensate for the position and transmission properties of each loudspeaker, the IR of each loudspeaker channel was convolved with a linear-phase equalization filter with 512 filter taps. The IRs were convolved live with the desired audio signals during the tests.

### Interleaved Acoustic Environments

The German matrix Test (Oldenburg sentence test, OLSA, Wagener et al., 1999) was used to obtain the speech perception scores or the SRTs in noise. The unique characteristic of the OLSA is its large number of test lists including 20 sentences each with inherent low memorability which ensures a high level of reproducibility (Steffens, 2017).

The OLSA was conducted in a closed mode, in which the test subjects themselves had to enter the perceived words of the sentence by pressing corresponding buttons on a touch screen monitor. One test list per acoustic environment was chosen randomly and contained 20 sentences each. After initial training to familiarize participants with the test material, all CI users had to accomplish the test twice, one run with activated ASC and one run with the standard microphone setting (randomized order between the subjects). The preprocessing features Automatic Sensitivity Control and Adaptive Dynamic Range Optimization preprocessing algorithms were always switched on. The frequency channel-based noise reduction algorithm (SNR-NR) was deactivated. All subjects used their daily map.

The SRT measurement was conducted adaptively with a fixed noise level of 60 dB SPL, with individual signal-to-noise ratio (SNR) calculation per acoustic environment. Initial SNR was set to +10 dB for each acoustic environment. Five acoustic environments encapsulated in one single OLSA task were tested:

1. **RVQ**

Reverberated (RV) speech in quiet (Q)

2. **RV-1N-CONT**

RV noise from 1 noise source (1N) at position N2 (Figure 2) with continuous speech-shaped (CONT) noise

3. **FF-1N-CONT**

Free field (FF) noise from 1N at position N2 (Figure 2) with CONT noise

4. **RV-4N-MOD**

RV noise from 4 noise sources (4N), placement N1-N4 (Figure 2), with decorrelated 4 Hz amplitude modulated (MOD) noises

5. **FF-4N-MOD**

FF noise from 4N, placement N1-N4 (Figure 2), with MOD noises

**Figure 3.**
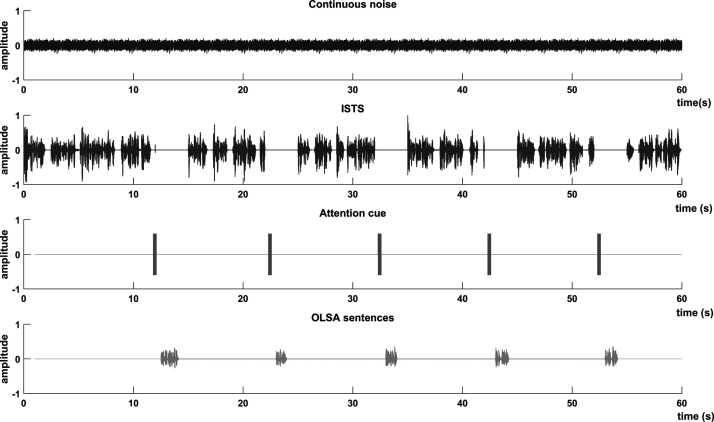
Playback Example of One Acoustic Environment Section (FF-1N-CONT) Visualized in Separated Audio Tracks: Continuous Noise, ISTS, Attention Cue and OLSA Sentence.

The IAEs speech perception test was finished after a full test list was completed for each acoustic environment. In the test, the acoustic environments were randomly changed section by section, with one acoustic environment section consisting of five OLSA sentences. The procedure for one acoustic environment section was as follows (also illustrated in Figure 3):

- Noise playback permanently until acoustic environment changes (not in RVQ)
- Initially 12 s of “International Speech Test Signal” (ISTS at 75 dB SPL, source S, 0°)
- Step 1-3 repeating five times: 1. Attention cue (horn signal, center frequency at 750 Hz, at 75 dB SPL, source S, 0°) 2. OLSA sentence (adaptive level, source S, 0°) 3.   ISTS until response confirmation (75 dB SPL, source S, 0°)

The time constant of 12 s for the initial ISTS was adopted from Mauger et al. (2014). Each individual OLSA sentence had a duration of around 2.5 s and was therefore too short to be processed by the ASC algorithm to find a speech reference. The additional ISTS signal was introduced to enable the adequate ASC setting. The positions of noise sources were mirrored for unilateral CI users if their implant was on the right side, or for bilateral subjects with a higher monosyllabic speech perception score in the right ear.

### Subjects

Sixteen unilateral (nine males, mean age: 58.9 ± 13.1 years), 16 bilateral (nine males, mean age: 60 ± 10.4 years) CI users, and 18 NH subjects (three males, mean age: 31.7 ± 6.6 years) took part in the study. All CI users had a listening experience with CI of at least 11 months and used cochlear nucleus 6 speech processors (CP900 Generation). Only patients with postlingual deafness were included in the study. Monosyllable score (Freiburg monosyllables, Hahlbrock, 1953) was least 50% at 65 dB (mean score: 70.9 ± 9.6% in unilateral and 76.4 ± 13.1% in bilateral CI group). (Supplementary material with information on demographics). Contralateral residual hearing in unilateral CI users was double-blocked by using ear plugs and a circumaural ear protection.

### Ethics Statement

The study was approved by the local institutional review board (reference number 164/16). Subjects gave their written consent and received financial compensation for their participation.

### Automated Scene Classification

The ASC algorithm automatically analyzes and classifies the acoustic environment. In the algorithm of the Cochlear speech processor, six predefined scenes are available for classification: speech in noisespeech in noisespeech in noisespeech in noise “*speech*,” “*noise*,” “*wind*,” “*quiet*,” and “*music*.” According to the classification, the algorithm selects a listening program that is linked to a suitable beamforming mode. The standard directional microphone pattern (beamformer with fixed sub-cardioid characteristic) is used in the scenes “quiet,” “speech,” and “music,” a beamformer with fixed supercardioid characteristic is selected in “noise” and an adaptive beamformer is activated when “speech in noise” is detected. Information about the selected program is displayed on the patient’s remote control.

### ASC Classification Pretest

A pretest was conducted to assess the classification results of the ASC algorithm in the five acoustic environments listed earlier. A test subject equipped with two CP900 sound processors was placed in the anechoic chamber and instructed to read out the classification results indicated on the display of the Cochlear CR230 remote control. The test subject performed a complete encapsulated OLSA test run with all IAEs. Table 1 shows the classification results of the ASC. When the acoustic environment RVQ was played back, classification was not consistent, which means that the algorithm could not perform a stable categorization. If the algorithm is unable to detect the acoustic environment, the prior categorization remains engaged. This means that for RVQ, the selected listening program depends on the categorization of the previous acoustic environment. For all other scenes, acoustic environment classification was consistent throughout the test.

**Table 1.** ASC Classification Results (Left and Right CI-Processor) Depending on Acoustic Environment (See Methods Section).

Acoustic environment	Left	Right
RVQ	–	–
FF-1N-CONT	SIN	S
RV-1N-CONT	SIN	SIN
FF-4N-MOD	S	S
RV-4N-MOD	SIN	SIN

*Note*. Classifications results were read from the remote control while the processors were placed on the ears of a pilot subject. Classification results: N = Noise, SiN = Speech in Noise, S = Speech, M = Music, SIL = Silence, – = No classification; Left = left ear; Right = right ear; RVQ = reverberated (RV) speech in quiet (Q); FF-1N-CONT = free field noise from 1N at position N2 with CONT noise; RV-1N-CONT = RV noise from 1 noise source (1N) at position N2 with continuous speech-shaped (CONT) noise; FF-4N-MOD = FF noise from 4N, placement N1-N4, with MOD noises; RV-4N-MOD = RV noise from 4 noise sources (4N), placement N1-N4, with decorrelated 4 Hz amplitude modulated (MOD) noises.

In acoustic environment FF-1N-CONT, the classification differs between the left and right processor. Ipsilateral to the noise source, *speech in noise* is classified. Contralateral to the noise source, *speech* is detected. For all other acoustic environments, classifications between the left and right processor were consistent. SNR or absolute level did not affect classification in the entire test run.

### Statistics

The statistical analysis was performed with SPSS 22 (IBM, Armonk, NY, USA). All data sets were tested for normal distribution using the Shapiro–Wilk test. Because for the majority of data sets normal distribution was not indicated, a Wilcoxon test (paired samples) or Mann–Whitney *U* -test (independent samples) was used. Multiple comparisons were compensated by Bonferroni correction. Comparisons with more than two groups were performed with a Kruskal–Wallis test. In the figures, significant results with *p *<* *.001 are marked with three asterisks (***), *p < *.01 is indicated by two asterisks (**), and *p < *.05 is marked by one asterisk (*).

All results are displayed as boxplots with the median indicated by a horizontal line inside the box. The lower boundary of the box is the first quartile, the upper boundary the third quartile, so that the entire box covers 50% of all data. Moreover, 1.5 times the interquartile range is represented as whiskers, while data outside the whiskers are classified as outliers that are displayed as circles. If a data point is more than three times the interquartile range outside the box, it is defined as an extreme outlier and marked as an asterisk.

## Results

### Speech Perception in Quiet

**Figure 4.**
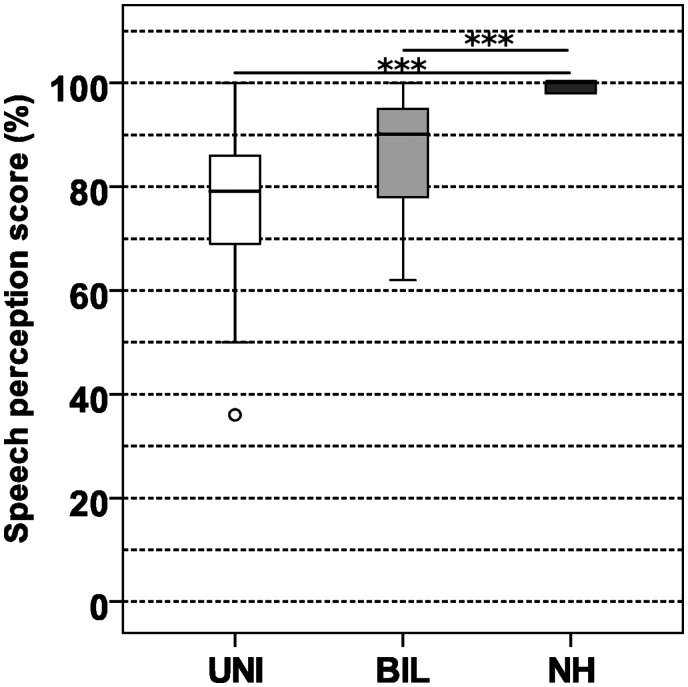
Speech Perception Score in Quiet (%) With Simulated Reverberation (Acoustic Environments RVQ) of UNI and BIL CI Users and NH Control Group. German OLSA matrix test, further details in text. Boxplot description see text. UNI = unilateral; BIL = bilateral; NH = normal hearing.

Figure 4 depicts boxplots with speech perception results obtained with the OLSA matrix test in the acoustic environment with speech in a simulated reverberant room (RVQ) for each subject group. CI users were tested in the standard microphone mode. The unilateral CI group achieved an average discrimination rate of 79%, the bilateral CI group 90%, and the NH group 100%. A significant effect of subject group was shown (χ^2^* = *28.009, *df = *2, *p < *.001). Pairwise comparison indicated no significant difference between unilateral and bilateral CI user groups (*U = *67.50, *Z =* −2.287, *p = *.063). A significant difference between unilateral CI users and NH (*U = *13.0, *Z =* −4.631, *p < *.001) and between bilateral CI users and NH (*U = *30, *Z =* −4.063, *p < *.001) was found.

### Mean IAE SRT

**Figure 5.**
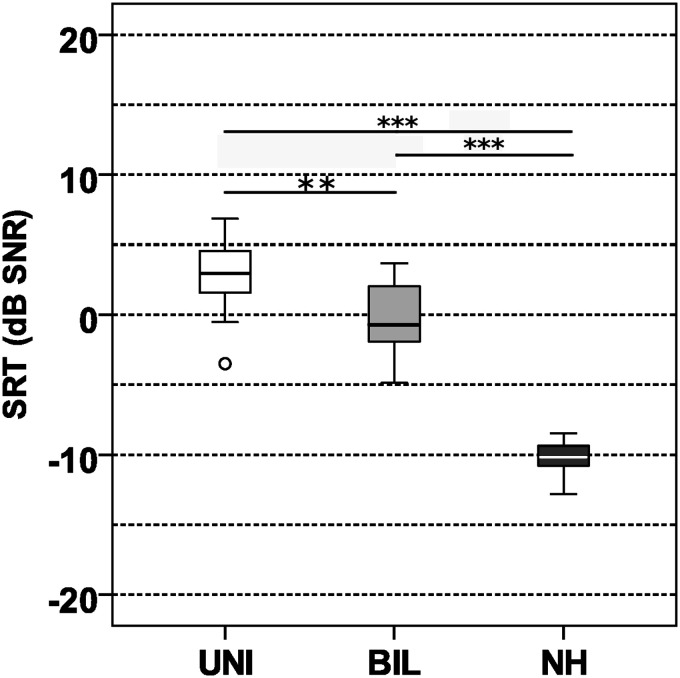
Mean-SRT Depending on Subject Group, Averaged Across Four Acoustic Environments With Additional Noise (FF-1N-CON, RV-1N-CON, FF-4N-MOD, RV-4N-MOD, See Text). The displayed results were obtained with standard microphone directionality (CI users). UNI = unilateral; BIL = bilateral; NH = normal hearing; Mean-SRT = mean speech reception threshold; SNR = signal-to-noise ratio.

Figure 5 shows OLSA mean Mean-SRT results for the two cohorts of CI users with processor settings in standard directionality (fixed sub-cardioid beamformer, ASC off) and for the NH group. SRTs obtained of each acoustic environment (continuous and modulated noise in FF and reverberation) were combined (averaged) to implement a measure for speech perception in multiple IAEs. A significant effect of subject group (χ^2^* = *36.932, *df* =* *2, *p < *.001) existed. Unilateral CI users had a mean SRT of 2.9 dB SNR, and bilateral CI users showed a significantly lower mean SRT of −0.8 dB SNR (*U = *48.50, *Z =*−2.997, *p = *.009). The mean SRT of the NH group was −10.2 dB SNR, which was significantly better than the result of unilateral (*U = *0.0, *Z =*−4.6968, *p < *.001) and bilateral CI users (*U = *0.0, *Z =*−4.6968, *p < *.001).

### Impact of Reverberation and Noise Modulation on SRTs

**Figure 6.**
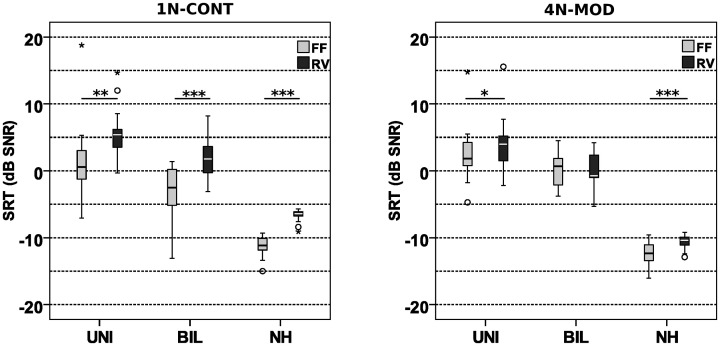
Impact of Reverberation and Subject Group on SRT. Left: OLSA SRTs, unilateral and bilateral CI group and NH listeners in the acoustic environment with additional noise 1 N-CONT, with and without reverberation. Right: OLSA SRTs of unilateral and bilateral CI group and NH listeners in acoustic environment with additional noise 4 N-MOD, with and without reverberation. UNI = unilateral; BIL = bilateral; NH = normal hearing; Mean-SRT = mean speech reception threshold; SNR = signal-to-noise ratio; FF = free field; RV = reverberation.

Figure 6 shows SRTs of unilateral CI users, bilateral CI users, and the NH group in acoustic environments in continuous noise (left panel) and modulated noise (right panel) with and without reverberation. All CI users used the standard microphone mode (ASC off). Speech perception in the cohort of unilateral CI users was negatively affected by reverberation in both noise conditions. They achieved a median SRT of 0.6 dB SNR in the FF acoustic environment with continuous noise (FF-1N-CONT). Compared to this condition, SRTs in reverberation (RV-1N-CONT) were significantly higher (5.4 dB SNR, *Z =*−3.051, *p = *.002). With modulated noise, the group of unilateral CI users had an SRT of 1.8 dB SNR in the FF acoustic environment (FF-4N-MOD) which increased significantly to 4 dB SNR in reverberation (RV-4N-MOD, *Z =*−2.224, *p = *.026).

In bilateral CI users, SRTs were significantly worse with reverberation in continuous noise. They had a median SRT of −2.5 dB SNR in the FF condition FF-1N-CONT, with reverberation (RV-1N-CONT), the SRT declined significantly to 1.8 dB SNR (*Z =*−3,517, *p < *.001). However, in modulated noise, reverberation did not significantly affect SRTs. In the respective FF environment (FF-4N-MOD), the group of bilateral CI users had an SRT of 0.7 dB SNR, with reverberation (RV-4N-MOD) the SRT was at −0.8 dB SNR (*Z =*−0.103, *p = *.918).

Reverberation negatively affected the NH listeners with both noise types. The SRT worsened significantly in continuous noise, it declined from −11.3 dB SNR (free field, FF-1N-CONT) to −6.5 dB SNR (reverberation, RV-1N-CONT, *Z =*−3.724, *p < *.001). With modulated maskers, the NH group had an SRT of −12.7 dB SNR in free field (FF-4N-MOD) which increased to −10.5 dB SNR with reverberation (RV-4N-MOD, *Z =*−3.550, *p < *.001). All subject groups showed a comparable decline of speech perception due to reverberation with continuous noise (unilateral: 4.8 dB SNR, bilateral: 4.3 dB SNR, NH: 4.8 dB SNR, mean amount of decline). With modulated noise, the distorting impact of reverberation was similar for unilateral CI users and NH. Speech perception declined on average by 2.2 dB SNR in both groups. However, the median SRT of the bilateral CI group surprisingly improved by 1.5 dB SNR, albeit not significantly.

### Effect of Subject Group

A significant group effect was shown for all acoustic environments which comprised noise (FF-1N-CONT: χ^2^* = *33.989; FF-4N-MOD: χ^2^* = *35.158; RV-1N-CONT: χ^2^* = *37.154; RV-4N-MOD: χ^2^* = *38.493; all *df *=* *2, all *p *<* *.001).

The post hoc comparisons results of all pairwise comparison are shown in Table 2. The largest SRT difference was found in acoustic environment RV-4N-MOD between NH and unilateral CI users. Here, the difference was 14.4 dB SNR. Nevertheless, some results of individual test persons clearly deviated from the mean results of their group. In condition FF-1N-CONT, bilateral CI user (Subject BI9) achieved an SRT of −13 dB, which was comparable to NH group results.

**Table 2.** *p* Values Indicating Significance Between Subject Groups for Each Tested Noise Acoustic Environment.

	FF-1N-CONT	RV-1N-CONT	FF-4N-MOD	RV-4N-MOD
UNI vs. BIL	*p* = .021	*p* < .001	*p =* .24	*p* = .005
NH vs. UNI	*p* < .001	*p* < .001	*p* < .001	*p* < .001
NH vs. BIL	*p* < .001	*p* < .001	*p* < .001	*p* < .001

*Note*. BIL = bilateral; UNI = unilateral; NH = normal hearing; FF-1N-CONT = free field noise from 1N at position N2 with CONT noise; RV-1N-CONT = RV noise from 1 noise source (1N) at position N2 with continuous speech shaped (CONT) noise; FF-4N-MOD = FF noise from 4N, placement N1-N4, with MOD noises; RV-4N-MOD = RV noise from 4 noise sources (4N), placement N1-N4, with decorrelated 4 Hz amplitude modulated (MOD) noises.

One subject (Subject Uni14) showed a relatively poor speech perception rate of less than 40% even in quiet with reverberated speech (RVQ). This subject consistently had the worst SRTs and is marked as an outlier in most analyses.

### Impact of Noise Setup in Acoustic Environments on Speech Perception

The impact of noise amplitude modulation and spatial configuration was assessed by comparing the absolute results of acoustic environments with continuous and modulated noise for each subject group. In the FF acoustic environment, all subject groups had a different outcome when the results of the noise setups were compared. Unilateral CI users showed no significant difference when the conditions with continuous and modulated noise were compared (*Z* = −1.706, *p = *.088). However, bilateral CI users had a significantly better SRT in the FF acoustic environment with continuous noise (FF-1N-CONT) compared to modulated noise (FF-4N-MOD, *Z* = −3.387*, p = *.001). In NH, significantly worse SRT was observed in the acoustic environment with continuous noise (FF-1N-CONT) compared to modulated noise in FF-4N-MOD (*Z* = −2.592, *p = *.010).

The impact of the noise setup in reverberation was similar for unilateral and bilateral CI users as well as for NH persons. In all subject groups, the result of the acoustic environment with continuous noise (RV-1N-CONT) was significantly worse than with modulated noise (RV-4N-MOD, unilateral: *Z* = −2.999, *p = *.003; bilateral: *Z* = −2.844, *p = *.004; NH: *Z* = −3.733, *p < *.001).

### Impact of ASC on SRTs

#### Mean IAE Improvement

Averaged across all four acoustic environments, median SRT calculated from the results of the group of unilateral CI users significantly improved by 2.4 dB SNR, from 2.9 dB SNR (ASC off) to 0.5 dB SNR when ASC was enabled (*Z* = −3.051, *p = *.002). Bilateral CI users improved their Mean-SRT by 1.3 dB SNR, from −0.8 dB SNR (ASC off) to −2.1 dB SNR (ASC on, *Z =*−2.947, *p = *.003).

#### FF Acoustic Environments

**Figure 7.**
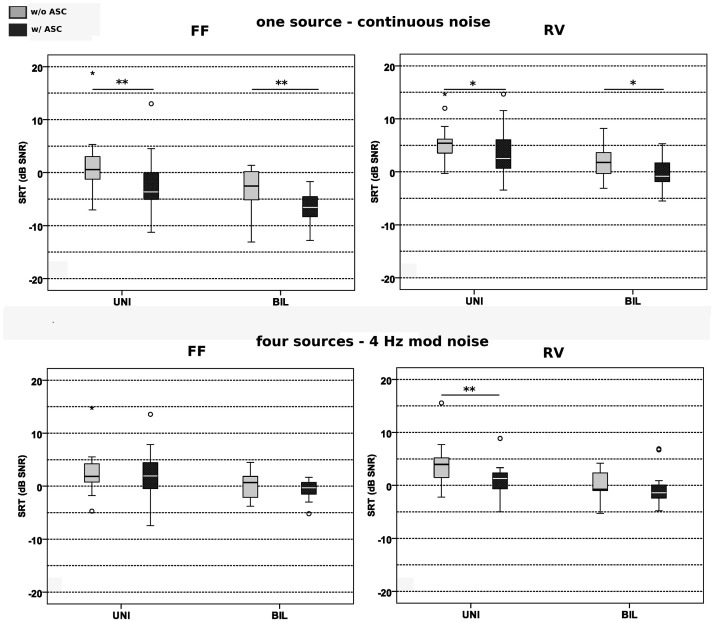
OLSA Matrix Test SRTs Depending on ASC Setting and Subject Group (UNI Unilateral, BIL Bilateral CI Users), and Acoustic Environment. Column left: free field anechoic conditions FF-1N-CONT and FF-4N-MOD, column right reverberated conditions: RV-1N-CONT and RV-4N-MOD. UNI = unilateral; BIL = bilateral; NH = normal hearing; Mean-SRT = mean speech reception threshold; SNR = signal-to-noise ratio; FF = free field; RV = reverberation.

Figure 7 shows SRT results depending on acoustic environment, determined with either enabled or disabled ASC setting (fixed standard microphone directional sensitivity). Results in modulated noise were poorer than in continuous noise. In the FF acoustic environment with continuous noise (FF-1N-CONT) a significant SRT improvement from 0.6 dB SNR (ASC off) to −3.6 dB SNR (SRT gain of 4.2 dB SNR) was found for the group of unilateral CI users with activated ASC algorithm (*Z =*−3.051, *p = *.002). Likewise, in the cohort of bilateral CI users, SRT improved significantly from −2.5 dB SNR (disabled ASC) to −6.6 dB SNR with enabling of ASC (*Z =*−3.051, *p = *.002, SRT gain 4.1 dB SNR).

With modulated noise, in free field (FF-4N-MOD) ASC did not affect performance significantly. The median SRT obtained in the cohort of unilateral CI users increased from 1.8 dB SNR (ASC off) to 2 dB SNR when ASC was activated (SRT difference 0.2 dB SNR). However, this difference was not significant (*Z =*−1.761, *p = *.078). The median group SRT of bilateral CI users differed by 1 dB SNR, it changed from 0.7 dB SNR to −0.3 dB SNR when ASC was activated, but again without significant difference (*Z =*−1.293, *p = *.196).

#### Reverberant Acoustic Environments

With reverberation SRTs were improved in both noise setups when ASC was activated. In the acoustic environment with continuous noise and reverberation (RV-1N-CONT), SRT improvement with activated ASC was smaller than in the corresponding FF acoustic environment. The group of unilateral CI users showed a significant improvement from 5.4 dB SNR (ASC off) to 2.5 dB SNR (ASC on, *Z =*−2.585, *p = *.010), the SRT difference was 2.9 dB SNR. Group results of bilateral CI users demonstrated also a significant SRT improvement of 2.6 dB (from 1.8 dB SNR [ASC off] to −0.8 dB SNR [ASC on], *Z =*−2.534, *p = *.011).

In contrast to the results obtained in acoustic environment with modulated noise in FF, the enabling of ASC improved median SRT results considerably in reverberant conditions. The median SRT gained in the cohort of unilateral CI users improved by 2.7 dB significantly from 4 dB SNR (ASC off) to 1.3 dB SNR when ASC was activated (*Z =*−2.898, *p = *.004). The median SRT calculated for the results of the cohort of bilateral CI users changed from −0.8 dB SNR (ASC off) to −1.4 dB SNR with enabled ASC. However, the effect of 0.6 dB was not significant (*Z =*−1.293, *p = *.196).

## Discussion

In this study, an experimental setup was presented, which enabled speech perception tests in IAEs. The test configuration offers a new approach to validate the effectiveness of ASC on speech perception in complex listening conditions. With the help of an application for simulating room acoustics, different listening situations were generated. A customized auralization system integrating a total of 128 loudspeakers allowed precise replication of early reflections in the horizontal plane. During one test run, acoustic environments with different noise characteristics (continuous and amplitude modulated noise), different spatial source arrangements (one noise source or four noise sources), and FF presentation or reverberation were presented. Cohorts of NH subjects and unilateral and bilateral CI users participated.

Compared to the Mean-SRT in the standard microphone mode, lower Mean-SRT thresholds resulted from the activation of ASC in bilateral and unilateral CI users. However, automated classification and program selection of some acoustic environments did not match the expected results. Thus, a benefit of ASC on speech perception could not be demonstrated in all individual acoustic environments. Speech perception of the test groups showed different effects on reverberation and type of noise.

### Automated Scene Classification

In this study, IAEs were presented during one single test run. Since the strength of ASC is to dynamically adapt the listening program, the IAE test setup is more suitable to investigate the impact of ASC on speech perception than test setups with static listening environments. In the IAE test, the ASC algorithm had to adapt to the different acoustic environments. The use of the introduced interleaved listening conditions is an important aspect since (a) misclassifications can potentially occur and (b) the classificator potentially remains in the previous state of classification. In previous studies that evaluated ASC, speech perception tests were conducted with different noise types and spatial arrangements (Mauger et al., 2014; Searchfield et al., 2018), which were tested sequentially. In the proposed IAE procedure, the variation of the listening environments during the test shows whether ASC also offers an advantage for speech perception in changing listening conditions.

Numerous previous studies have shown that beamformers can significantly improve speech perception in CI users (Büchner et al., 2014; Honeder et al., 2018; Spriet et al., 2007). However, CI users hesitate to switch to appropriate listening programs (Gifford & Revit, 2010). ASC minimizes user interaction as listening programs are activated and deactivated automatically. The reduced necessity to manually change programs can lead to significantly improved SRTs in complex listening scenarios. However, the benefit of ASC on speech perception depends on a sensible classification in conjunction with the acoustic and room acoustic properties. The pretest results revealed some misclassifications of the ASC algorithm. In condition RVQ (speech with reverberation), ASC did not classify any specific listening environment and remained in its previous state. With one continuous noise source in FF, the left and right processor showed different ASC classifications. This leads to an asymmetric program selection and, therefore, potential deterioration of binaural cues. It demonstrates that an adverse effect of ASC may occur compared to manual program selection. Consequently, manual selection of the dedicated speech-in-noise program can be advantageous over the use of ASC in static listening environments. The speech-in-noise program constantly uses a beamformer and should, therefore, lead to the best possible speech perception outcome in noisy environments.

### Speech Perception in IAEs With ASC

Both CI groups showed an improved Mean-SRT using ASC compared with the standard microphone sensitivity (amount of improvement unilateral CI 2.4 dB SNR, bilateral CI 1.3 dB SNR). Mauger et al. (2014) reported larger SNR improvements with ASC in a mixed group of unilateral and bilateral CI users in different noise scenarios (speech weighted noise: 3.9 dB SNR improvement, babble noise: 3.5 dB SNR improvement). However, an additional noise reduction algorithm was also activated in the ASC condition. Therefore, the resulting benefit in their study includes both, the benefit of ASC and noise reduction. Gifford and Revit (2010) tested the benefit of a manually activated adaptive beamformer on speech perception in a simulated diffuse background noise of a restaurant presented with the R-Space (Revit et al., 2007). With the activated adaptive beamformer, the subjects achieved a mean SRT of 6.6 dB SNR, without the beamformer, the SRT was 10.2 dB SNR.

In both aforementioned studies, the benefit with ASC-linked or manually activated beamformer was higher than the Mean-SRT improvement with ASC in the current study. However, in this study, the benefit of ASC varied significantly with the acoustic environment. Especially with reverberation and/or modulated noise, the beneficial effect of ASC degraded significantly. The Mean-SRT covers several noisy listening situations and is therefore more relevant for the patient than the result of a single test environment. In addition, the presented study was the first study that took the transition from one to another acoustic condition into account which is more challenging for the ASC algorithm.

### Speech Perception in FF With ASC

The strengths as well as the weaknesses of ASC became apparent in FF acoustic environments. The highest improvement in SRT was found with a single (static envelope) noise source in the absence of reverberation (FF-1N-CONT). According to the results of the pretests, the ASC of the processor contralateral to the noise source classified *speech in quiet* in the FF-1N-CONT condition, while the ipsilateral ASC switched to *speech in noise*. Despite setting two different listening programs (left/right in bilateral CI), the SNR improvement with ASC enabled was similar between unilateral and bilateral CI users (4.2 dB and 4.1 dB, respectively). Thus, bilateral mismatching classification did not have a negative effect compared to unilateral listening.

Pretest results revealed that ASC classification of speech together with multiple modulated noise sources (condition FF-4N-MOD) erroneously detected *speech*, even though *speech in noise* was expected. This means that the processors used the standard (fixed sub-cardioid) beamformer instead of changing to the adaptive beamformer (setting for *speech in noise*). Potentially for this reason, a significant ASC benefit was absent.

### Speech Perception in Reverberation With ASC

A smaller ASC benefit was observed with reverberation in the condition with one continuous noise source. Since masking noise is more diffuse with reverberation, the effect of the beamformer is reduced, as the strongest attenuation appears in the rear hemi field. Nonetheless, SRTs still improved by 2.9 and 2.6 dB SNR (unilateral and bilateral CI users, respectively) with activated ASC.

With modulated noise (environment RV-4N-MOD), there was a positive impact of ASC on speech perception. ASC enabled *speech in noise* and therefore activated the adaptive beamformer. Consequently, in the unilateral CI cohort the beneficial effect of ASC amounted to 2.7 dB SNR, but bilateral CI users only demonstrated a nonsignificant improvement of 0.6 dB SNR with activated ASC. One reason for this discrepancy could be the head shadow effect. In unilateral CI users, noise source N4 was on the side contralateral to the processor (Figure 2, Methods section). Therefore, less impact on speech perception was expected compared to bilateral CI users. Noise of source N4 can hardly be reduced by the adaptive beamformer, since it mainly works in the rear half field (Spriet et al., 2007).

### Potential Limitations of the Test Setup

#### Matrix Sentence Test

In this study, the determination of individual SRTs is based on the German matrix sentence test (OLSA). The IAE test focused on testing different noise conditions with listening environments that include multiple acoustic and room acoustic aspects. For instance, noise modulation characteristics were comparable to the modulation of spoken language and reverberation was modeled to represent the acoustics of a real auditorium. Therefore, the reproduction of each noise condition can at least somehow approximate a certain listening situation in everyday life. On the other hand, the used speech signal and test procedure are not related to real everyday life conversation. The matrix test uses nonsense sentences that all have the same structure (name, verb, numeral, adjective, and noun). In contrast to other sentence tests with open set speech material, the subject has a priori knowledge of the sentence structure. Thus, the matrix sentence test does not represent real-life communication. Applying open set speech material could potentially reveal additional effects arising from the acoustic environment and from signal preprocessing. For example, cognitive factors supporting speech perception in everyday life are not captured by the proposed speech perception measurement procedure. The matrix sentence test is well suited for testing differences between speech processor settings; however, it is not a measure of everyday communication ability.

#### Recording of ASC Classification

Since ASC classification results were not controlled during the actual tests run, there is no certainty whether the classifications of the test subjects’ processors differed from those of the preliminary investigation. It is possible that the processors carried different classifications due to individual circumstances, such as reflections on the test subject’s body, or that switching processes of ASC took longer. Future studies should consider the recording of classifications during the test run.

#### Room Simulation

In the diagnosis and therapy of hearing disorders, test procedures under FF conditions are usually performed. Often setups with only one or two sound sources (loudspeakers) are used, which limits the possible directions of speech and background noise. However, challenges for CI users include situations like family celebrations or visiting the restaurant. Therefore, more complex setups for speech perception tests in clinical routine should be available. Especially the use of amplitude-modulated maskers in combination with reverberation and spatial separation of target and noise sources are informative.

Room simulation offers an efficient way to change the acoustic properties of a virtual room. By using room simulation, three-dimensional room models can be created, imported, and varied as desired. Once the framework for simulation and sound reproduction is established, the method is effective compared to previously used methods, such as the covering of real rooms with different materials (Kidd et al., 2005; Marrone et al., 2008). Earlier applications of virtual room models tested only speech perception in CI users in quiet (Kressner et al., 2018). Speech perception in noise and/or reverberation in CI users has rarely been studied. On the one hand, virtual reality labs at universities have typically less access to subjects with CIs. On the other hand, loudspeaker setups are very complex and therefore not available in many ENT clinics. Therefore, a reduction of complexity is desirable. Future studies should evaluate if a simpler setup with 8 to 12 loudspeakers could potentially be sufficient to extend current clinical test setups for testing speech perception in complex noise conditions. With the current setup with 128 loudspeakers, the study is not reproducible for other clinics.

### Speech Perception: NH Versus CI Users

In this study, SRT difference between bilateral CI users and NH control group averaged across acoustic environments was 9.4 dB SNR, and more than 13 dB SNR between unilateral CI users and the NH control group. These large SRT differences reflect the significant perceptual difficulties of CI users in noisy listening situations. In measurement conditions where spatial release from masking is advantageous, unilateral CI patients are particularly negatively affected by the lack of second ear coverage and of binaural hearing.

### Impact of Noise Type and Spatial Configuration

The NH group demonstrated an improved SRT with amplitude modulated maskers in FF compared to continuous noise (benefit 1.4 dB SNR). In contrast, speech perception in both CI groups deteriorated in FF with amplitude modulated maskers compared to continuous noise (unilateral CI: decrement 1.2 dB SNR, bilateral CI: decrement 3.2 dB SNR). Previous studies have shown that persons with NH can listen in short dips of amplitude modulated maskers (Fastl, 1987). During those short gaps in the masking noise, the SNR is more positive and speech perception is improved. This so-called *glimpsing effect* (Bronkhorst & Plomp, 1992; Cooke, 2006; Festen, 1990) does not exist in CI users with electric stimulation (Weissgerber et al., 2015, 2017; Zirn et al., 2016) and with combined electric-acoustic stimulation (Rader et al., 2013). On the contrary, even a deterioration of speech perception in CI users in amplitude modulated noise compared to continuous noise was observed. These findings were confirmed by the results of this study, where bilateral CI users in particular showed large detrimental effects.

### Impact of Reverberation

Previous studies that used the direct input of the CI to present reverberant stimuli (Hazrati & Loizou, 2012; Weissgerber et al., 2016) showed that when noise and reverberation co-exist, both degrade listening performance. The results of this study showed that the influence of reverberation depends on both, the temporal envelope of the noise and the spatial configuration of the noise sources. In the acoustic environment with continuous noise, the impact of reverberation was similar in all tested groups. NH had an adverse effect of 4.8 dB SNR, unilateral CI users of 4.8 dB SNR and bilateral CI users of 4.3 dB SNR. Compared to FF presentation, spatial release from masking (SRM) is degraded with reverberation. Diffuse reflections reduced the head shadow effect, resulting in reduced SRM. Eichenauer et al. (2020) reported on the effect of reverberation on SRM. They observed that SRM reduced from 6.8 dB in FF to 1.3 dB in reverberation. Since the interaural level differences were reduced by up to 10 dB, the authors concluded that the help of the head shadow effect in reverberation is very small.

With multiple sources of amplitude modulated noise (condition 4 N-MOD), the effect of reverberation on SRTs was ambiguous. NH and unilateral CI users had a reduction of speech perception of 2.2 dB SNR, while the SRT of bilateral CI users improved by 1.5 dB SNR. One explanation for these conflicting results could be the effect of reduced amplitude modulation depth by reverberation. Temporal gaps are filled with reverberated signals, and the detrimental effect of masker modulations on SRT is smaller.

In people with NH, the detrimental effect of reverberation on speech perception is higher in continuous noise than in modulated noise. In modulated noise, gap listening is still possible despite reverberation. In addition, four spatially separated noise sources are used in the conditions with amplitude modulated noise. This means that the sounds are already presented diffusely and the effect that noise is more diffuse due to reflected sounds is smaller.

## Conclusion

The proposed interleaved listening environments (IAE) procedure is a useful tool to evaluate speech perception in listening environments of different complexity. The application of the IAE method showed that a beneficial effect of ASC on SRTs can be demonstrated, even in changing environments. However, the benefit provided by ASC was depending on the acoustic environment. Larger benefits were observed in the continuous noise acoustic environment. The effect of ASC was smaller in reverberation and even absent in modulated noise.

The current results demonstrate that the IAE-procedure is an effective tool to assess the effect of signal processing on SRTs in complex acoustic environments.

## Supplemental Material

sj-xlsx-1-tia-10.1177_23312165211014118 - Supplemental material for Interleaved Acoustic Environments: Impact of an Auditory Scene Classification Procedure on Speech Perception in Cochlear Implant Users Click here for additional data file. Supplemental material, sj-xlsx-1-tia-10.1177_23312165211014118 for Interleaved Acoustic Environments: Impact of an Auditory Scene Classification Procedure on Speech Perception in Cochlear Implant Users by Anja Eichenauer, Uwe Baumann, Timo Stöver and Tobias Weissgerber in Trends in Hearing
